# Supplementary material for: Role and mechanism of NCAPD3 in promoting malignant behaviors in gastric cancer
Source: Front Pharmacol. 2024 Apr 22;15:1341039. doi: 10.3389/fphar.2024.1341039 (PMC11070777; doi:10.3389/fphar.2024.1341039)
Supplement: Supplementary file 11 [file DataSheet2.ZIP › GSEA/immunologic/my_analysis.Gsea.1599462003817/GSE43863_LY6C_INT_CXCR5POS_VS_LY6C_LOW_CXCR5NEG_EFFECTOR_CD4_TCELL_DN.html]

Details for gene set GSE43863\_LY6C\_INT\_CXCR5POS\_VS\_LY6C\_LOW\_CXCR5NEG\_EFFECTOR\_CD4\_TCELL\_DN[GSEA]

|  || Dataset | filtered\_dataset.sample\_info.cls#WT\_versus\_NCAPD3\_MUT |
| Phenotype | sample\_info.cls#WT\_versus\_NCAPD3\_MUT |
| Upregulated in class | NCAPD3\_MUT |
| GeneSet | GSE43863\_LY6C\_INT\_CXCR5POS\_VS\_LY6C\_LOW\_CXCR5NEG\_EFFECTOR\_CD4\_TCELL\_DN |
| Enrichment Score (ES) | -0.5694855 |
| Normalized Enrichment Score (NES) | -2.490769 |
| Nominal p-value | 0.0 |
| FDR q-value | 0.014169139 |
| FWER p-Value | 0.117 |
Table: GSEA Results Summary

  

Fig 1: Enrichment plot: GSE43863\_LY6C\_INT\_CXCR5POS\_VS\_LY6C\_LOW\_CXCR5NEG\_EFFECTOR\_CD4\_TCELL\_DN      
 Profile of the Running ES Score & Positions of GeneSet Members on the Rank Ordered List

  

| SYMBOL | TITLE | RANK IN GENE LIST | RANK METRIC SCORE | RUNNING ES | CORE ENRICHMENT || 1 | 1119 | CHKA | 670 | 0.390 | -0.4410 | No |
| 2 | 6303 | SAT1 | 850 | -0.233 | -0.5456 | Yes |
| 3 | 3309 | HSPA5 | 876 | -0.288 | -0.5340 | Yes |
| 4 | 6520 | SLC3A2 | 878 | -0.290 | -0.5050 | Yes |
| 5 | 9414 | TJP2 | 916 | -0.345 | -0.4961 | Yes |
| 6 | 9388 | LIPG | 937 | -0.361 | -0.4734 | Yes |
| 7 | 55591 | VEZT | 973 | -0.389 | -0.4587 | Yes |
| 8 | 3516 | RBPJ | 1021 | -0.419 | -0.4494 | Yes |
| 9 | 25976 | TIPARP | 1063 | -0.454 | -0.4323 | Yes |
| 10 | 7204 | TRIO | 1120 | -0.494 | -0.4218 | Yes |
| 11 | 1263 | PLK3 | 1134 | -0.502 | -0.3796 | Yes |
| 12 | 2152 | F3 | 1188 | -0.559 | -0.3603 | Yes |
| 13 | 837 | CASP4 | 1245 | -0.623 | -0.3366 | Yes |
| 14 | 3892 | KRT86 | 1313 | -0.724 | -0.3104 | Yes |
| 15 | 84959 | UBASH3B | 1338 | -0.776 | -0.2480 | Yes |
| 16 | 2650 | GCNT1 | 1342 | -0.781 | -0.1700 | Yes |
| 17 | 6541 | SLC7A1 | 1388 | -0.981 | -0.1017 | Yes |
| 18 | 7462 | LAT2 | 1399 | -1.138 | 0.0079 | Yes |
Table: GSEA details [plain text format]

  

Fig 2: GSE43863\_LY6C\_INT\_CXCR5POS\_VS\_LY6C\_LOW\_CXCR5NEG\_EFFECTOR\_CD4\_TCELL\_DN      
 Blue-Pink O' Gram in the Space of the Analyzed GeneSet

  

Fig 3: GSE43863\_LY6C\_INT\_CXCR5POS\_VS\_LY6C\_LOW\_CXCR5NEG\_EFFECTOR\_CD4\_TCELL\_DN: Random ES distribution      
 Gene set null distribution of ES for **GSE43863\_LY6C\_INT\_CXCR5POS\_VS\_LY6C\_LOW\_CXCR5NEG\_EFFECTOR\_CD4\_TCELL\_DN**
